# Trichovariability in rhizosphere soil samples and their biocontrol potential against downy mildew pathogen in pearl millet

**DOI:** 10.1038/s41598-021-89061-2

**Published:** 2021-05-04

**Authors:** Boregowda Nandini, Hariprasad Puttaswamy, Ramesh Kumar Saini, Harischandra Sripathy Prakash, Nagaraja Geetha

**Affiliations:** 1 Department of Studies in Biotechnology, University of Mysore, Manasagangotri, Mysuru, Karnataka 570 006 India; 2 Centre for Rural Development and Technology, Indian Institute of Technology Delhi, New Delhi, India; 3 Department of Crop Science, Konkuk University, Seoul, 143-701 Republic of Korea

**Keywords:** Applied microbiology, Fungi, Microbiology

## Abstract

The present work is aimed to examine the genetic variability and the distribution pattern of beneficial *Trichoderma* spp. isolated from rhizosphere samples and their mode of action in improving the plant health. A total of 131 suspected fungi were isolated from the rhizospheric soil and 91 isolates were confirmed as *Trichoderma* spp. *T. asperellum* and *T. harzianum* were found high in the frequency of occurrence. Genetic diversity analysis using RAPD and ISSR revealed the diverse distribution pattern of *Trichoderma* spp. indicating their capability to adapt to broad agroclimatic conditions. Analysis of genetic diversity using molecular markers revealed intra-species diversity of isolated *Trichoderma* spp. The frequency of pearl millet (PM) root colonization by *Trichoderma* spp. was found to be 100%. However, they showed varied results for indole acetic acid, siderophore, phosphate solubilization, β-1,3-glucanase, chitinase, cellulase, lipase, and protease activity. Downy mildew disease protection studies revealed a strong involvement of *Trichoderma* spp. in direct suppression of the pathogen (mean 37.41) in the rhizosphere followed by inducing systemic resistance. Our findings highlights the probable distribution and diversity profile of *Trichoderma* spp. as well as narrate the possible utilization of *Trichoderma* spp. as microbial fungicides in PM cultivation across different agroclimatic zones of India.

## Introduction

Pearl millet (*Pennisetum glaucum* (L.) R. Br.) (PM) is one of the most significant millet cultivated across semi-arid regions of the world. India is one of the largest producers of PM in the world, with approximately 7 million ha (Mha) area under cultivation. Rajasthan is the highest PM producing state within the country followed by Maharashtra, Gujarat, Uttar Pradesh, and Haryana, it accounts for more than 90% of PM acreage in the country. PM is the fourth most extensively cultivated crop after rice, wheat, and maize in India. It occupies an area of 6.93 Mha with an average production of 8.61 million tonnes with a productivity of 1243 kg/ha during 2018–2019^1^. It is the staple diet for the poorest people, and its stover is used as livestock feeds in rural India. As PM cultivation is carried out on marginal land, its productivity is always lesser than expected. Additionally, continuous application of synthetic fertilizers and pesticides in this region leads to reduced soil health by decreasing the population of native microbes and releasing organic carbon^2,3^. On the other hand, downy mildew (DM) of PM caused by *Sclerospora graminicola* is a widespread devastating disease reported to cause substantial annual yield losses in India^4–6^. The annual grain yield loss due to DM is estimated to 20–40%, however, the losses are much higher under unfavorable conditions of high relative humidity, moderate temperature, and extensive use of the same cultivar^7,8^. DM disease has been managed conventionally by seed priming with fungicide Apron 35 SD and foliar spray with Ridomil MZ^9^.

Agriculture relatively relies on close associations between plants and the microbes that live in association with plant roots. These rhizosphere microorganisms are distinct from other microbial communities with their high biocontrol potential and they are more important to the plant for nutrient uptake and to boost plant health against pathogen^10^. To achieve this, it is important to ascertain the functional and structural diversity of desired microbes within particular agroclimatic conditions. *Trichoderma* has been widely employed and studied as a biocontrol agent against a wide range of phytopathogens including bacteria^11,12^, fungi^13–16^, oomycetes^17,18^, and nematode^19,20^ in a wide range of crops and climatic conditions. In India, approximately 250 commercial formulations of *Trichoderma* spp. are being used^21,22^, and identification and characterization of new species for their potential biocontrol properties are under progress^23^. *Trichoderma* can reduce the severity of plant diseases either by directly inhibiting the growth and establishment of phytopathogens or by inducing systemic resistance (ISR) in the host plant^22,24,25^. Mechanism suggested to be involved in the biocontrol activity of *Trichoderma* spp. are antibiosis, lysis, competition, and mycoparasitism^26,27^. *Trichoderma* are capable to activate both SAR and ISR mechanism in plant hosts^28^. *Trichoderma* spp. are identified for their ability to produce bioactive secondary metabolites, including polyketides, terpenoids, alkaloids and peptaibols^29^. SMs secreted by various *Trichoderma* spp. own the potential to inhibit the growth of important plant pathogenic microrganisms. *T. harzianum* control the growth of *Clavibacter michiganensis* subsp. *michiganensis* by producing Lysosime^30^. *T. harzianum* T23 inhibit the growth of *Erwinia amylovora* and *C. michiganensis* in vitro by producing viridiofungin A^31^. Different peptaibols such as Trichokonin VI, VII and AVIII were identified in *Trichoderma* spp., which are the cause of the suppression of plant pathogens growth^32^. *Trichoderma* spp. are known to produce enzymes like chitinase and glucanase which can degrade fungal and oomycetous pathogen cell walls and also reported to produce antibiotics^33^. Further, they are known to produce phytohormones such as gibberellic acid and indole acetic acid (IAA), and cycling soil nutrients, thereby playing a crucial role in enhancing soil organic carbon and improving the soil configuration and fertility. Together with all these properties of *Trichoderma* spp. are involved in improving plant growth and health^34–36^.

The prospect of influencing crop rhizospheric microbial inhabitants by inoculating useful microorganism(s) to enhance plant growth and health has revealed substantial assurance in greenhouse and laboratory studies; however, variable responses have been noticed in the field studies^37^. The main reason behind the reduced efficiency of biocontrol agents under field conditions is their ability to adapt to local biotic and abiotic environmental conditions. To understand this phenomenon, it is necessary to study the geographical distribution pattern of biocontrol agents in the rhizosphere. Hence, the present investigation was undertaken to study (1) the distribution and genetic diversity patterns of *Trichoderma* spp. in PM growing regions of India, (2) the characterization of *Trichoderma* for their beneficial traits, and (3) the potential of *Trichoderma* to improve the host plant growth and suppress DM disease in PM.

## Results

**Figure 1:**
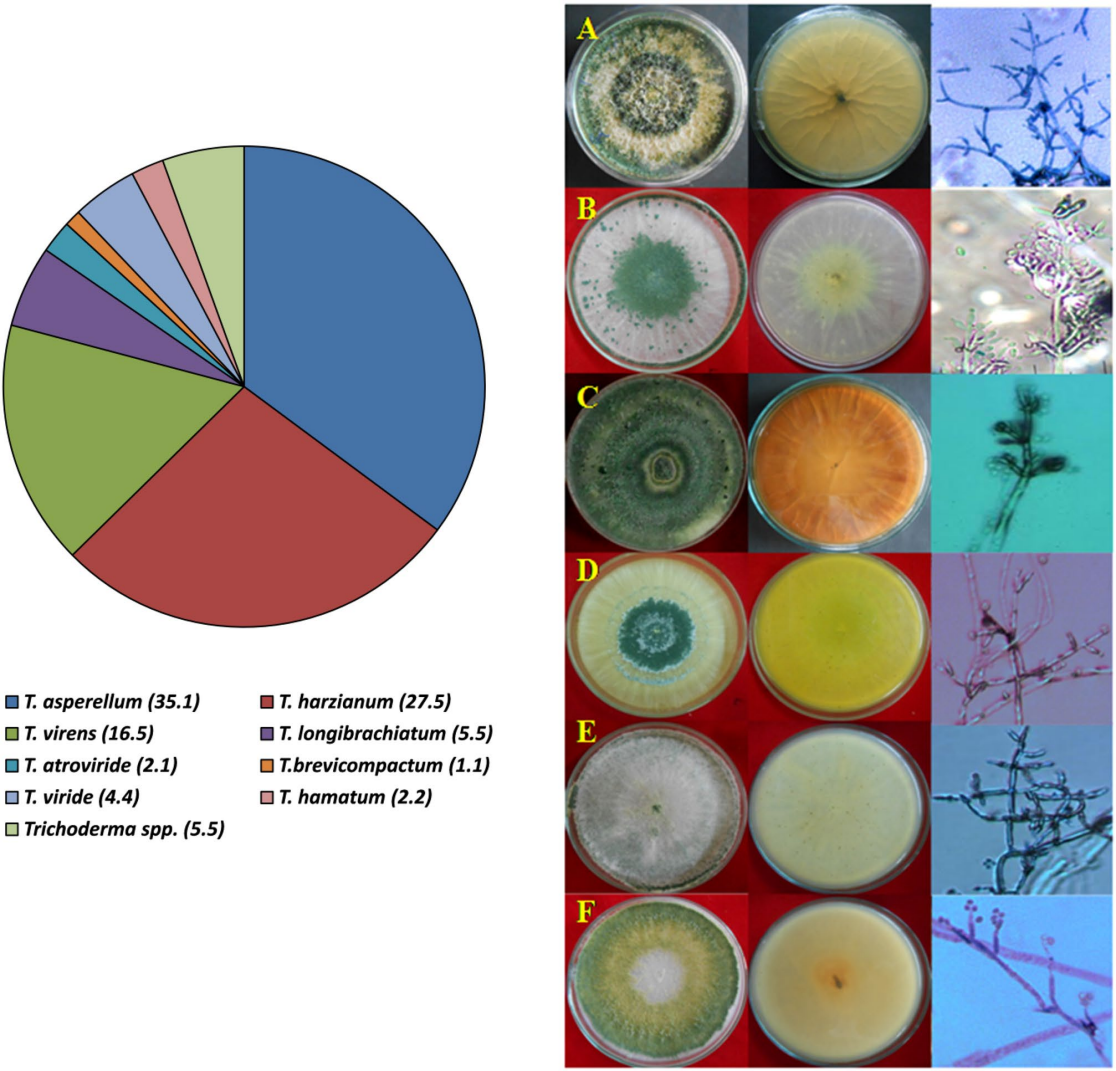
Diversity of *Trichoderma* spp. across different agroclimatic zones of India. The values in the parenthesis represents % *Trichoderma*. A—*T. asperellum*; B—*T. harzianum*; C—*T. virens*; D—*T. longibrachiatum*; E—*T. atroviride* and F—*T. brevicompactum*.

Suspected 131 fungal were isolated from 193 soil samples collected from 58 districts distributed across eight agroclimatic zones (Supplementary Fig. S1). They were morphologically characterized by growing on PDA plates. The isolates recorded wide morphological characters. Colonies of *Trichoderma* spp. appear initially w.hitish wooly and become compact as they grow. During conidia formation, they turn blue-green or yellow-green color. Some isolates grow in the form of concentric rings. The reverse is pale greenish, tan, and yellowish in different *Trichoderma* spp. (Fig. 1). Further the phialides, conidial arrangement, and conidial morphology varied among the isolates. The identity of the isolates was further confirmed by analyzing the ITS sequence (Supplementary Table S1). Collectively, these isolates were identified as *T. asperellum* (35.1%), *T. harzianum* (27.5%), *T. virens* (16.5%), *T. longibrachiatum* (5.5%), *T. atroviride* (2.1%), *T. brevicompactum* (1.1%), *T. viride* (4.4%), *T. hamatum* (2.2%), and five isolates (5.5%) were only identified up to genus level (Fig. 1). Among 131 *Trichoderma* isolates, 91 species (after removing duplicates and other fungal isolates), which are decisively identified, based on morphological and molecular profiles were selected for further studies. ITS sequence of *Trichoderma* isolates was submitted to the National Center for Biotechnology Information (NCBI, Bethesda, Maryland, USA) database, and an accession number was obtained. Distribution analysis revealed that *Trichoderma* spp. are widely distributed across different agroclimatic regions of India. Among the identified isolates, *T. asperellum* and *T. harzianum* were found in all agroclimatic regions. Whereas, *T. brevicompactum* was recorded only in soil samples collected from the state of Uttar Pradesh (Supplementary Table S1).

RAPD was carried out by using 20 sets of random primers. All the primers formed reproducible and polymorphic bands and were further selected for screening. In the size range of 0.1–5.0 kb, 97 bands were scored, with an average of 4.85 bands per primer. Among these bands, 51 were polymorphic in nature with 52.58% polymorphisms (Table 1). RAPD-7 seemed to be most proficient with 87.5% polymorphism compared to others. The PIC values were recorded in the range from 0.451 (RAPD-3) to 0.894 (RAPD-7) with average heterozygosity (mean of PIC) of 0.718. A representative illustration of RAPD profile obtained by RAPD-7 primer in the diversity studies of 91 *Trichoderma* spp. is shown in the supplementary Fig. S2.

**Table 1:** Summary of genetic diversity obtained by RAPD primers.

Sl. no.	Primer name	Primer sequence (5′–3′)	Tm value of primer (°C)	Polymorphic bands/total no of bands	PIC*
1	OPE-1	CCCAAGGTCC	37.3	3/5	0.750
2	OPE-2	GGTGCGGGAA	44.9	1/3	0.664
3	OPE-3	CCAGATGCAC	29.2	1/2	0.451
4	OPE-4	GTGACATGCC	28.4	5/7	0.859
5	OPE-5	TCAGGGAGGT	31.5	2/4	0.742
6	OPE-6	AAGACCCCTC	30.1	1/6	0.785
7	OPE-7	AGATGCAGCC	33.6	7/8	0.894
8	OPE-8	TCACCACGGT	34.3	4/5	0.762
9	OPE-9	CTTCACCCGA	34.9	2/7	0.866
10	OPE-10	CACCAGGTGA	29.1	1/5	0.756
11	OPE-11	GAGTCTCAGG	25.0	2/6	0.816
12	OPE-12	TTATCGCCCC	39.8	1/2	0.467
13	OPE-13	CCCGATTCGG	44.4	4/7	0.861
14	OPE-14	TGCGGCTGAG	42.4	5/7	0.842
15	OPE-15	ACGCACAACC	34.8	3/5	0.798
16	OPE-16	GGTGACTGTG	21.4	1/3	0.470
17	OPE-17	CTACTGCCGT	29.5	2/6	0.801
18	OPE-18	GGACTGCAGA	28.3	1/2	0.455
19	OPE-19	ACGGCGTATG	36.2	2/3	0.568
20	OPE-20	AACGGTGACC	32.7	3/4	0.749
	Average			51/97	0.718

*PIC* polymorphic information content.

**Figure 2:**
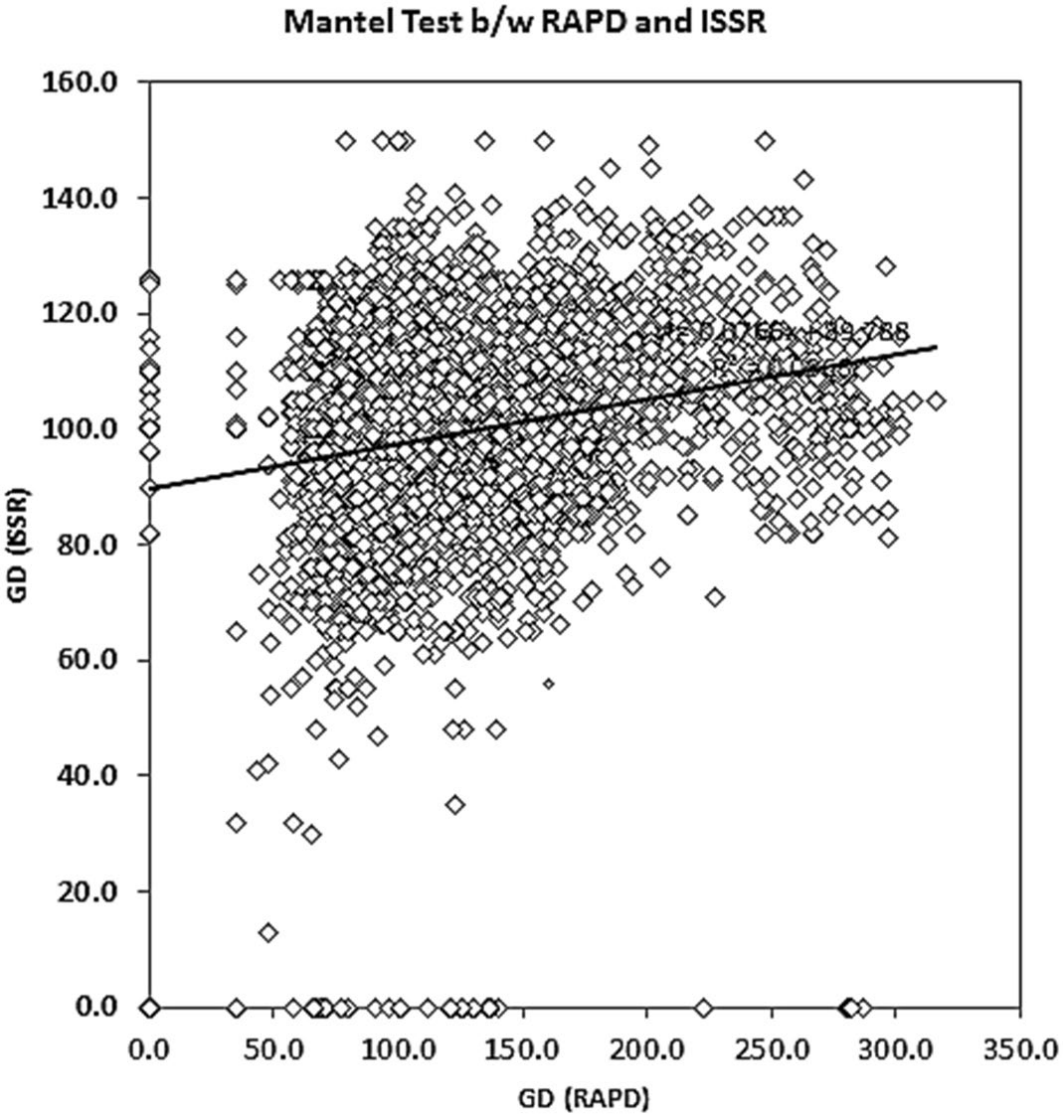
Mantel test for the 91 *Trichoderma* spp. representing the genetic distance between RAPD and ISSR primers.

**Figure 3:**
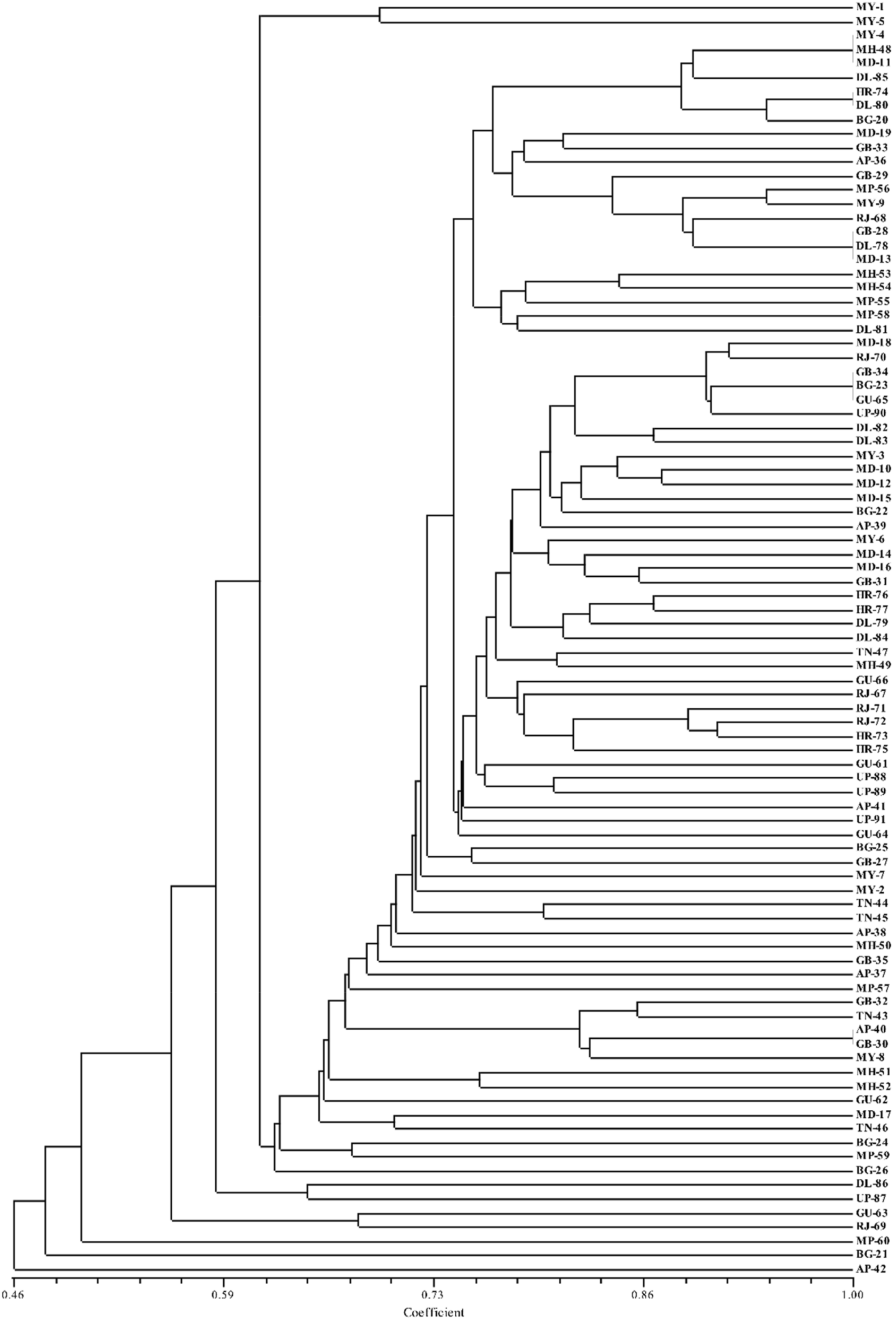
UPGMA cluster analysis showing the relationship and diversity among 91 *Trichoderma* isolates produced by RAPD and ISSR primers. Bootstrap values is > 50% from 1000 replications.

**Figure 4:**
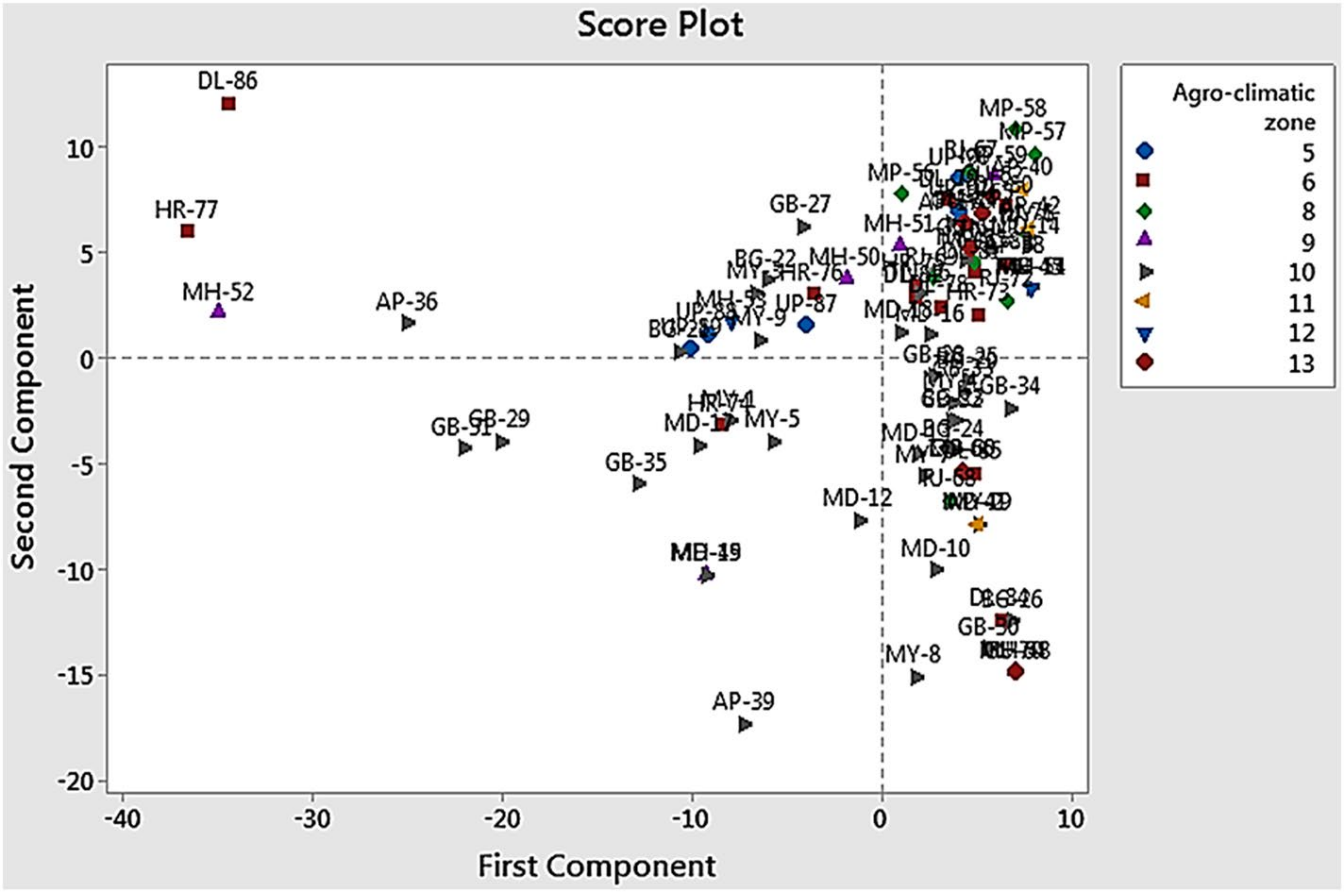
Principal component analysis of the distribution pattern of *Trichoderma* across different agroclimatic zones based on data generated through RAPD and ISSR analysis.

In ISSR assays, 12 primers showed polymorphic and reproducible bands. From the 91 isolates of *Trichoderma* spp., 54 bands were scored in the size range of 0.1–5 kb, with an average of 4.5 bands per primer. Of these 54 bands, 23 were polymorphic with 42.59% polymorphism (Table 2, Supplementary Fig. S3). ISSR-5 was found to be most efficient with 75.0% polymorphism compared to other primers. The PIC values were observed in the range from 0.453 (ISSR-6) to 0.874 (ISSR-4) with mean heterozygosity (average of PIC) of 0.704. However, in the mantel test (Fig. 2), a weak correlation (R^2^ = 0.095) was observed among genetic distance analyzed by RAPD and ISSR data of the *Trichoderma* isolates. The Unweighted Pair-Group Methods with Arithmetic Average (UPGMA) cluster analysis among 91 *Trichoderma* isolates produced by RAPD and ISSR primers is represented in Fig. 3. Principle coordinated analysis (PCA) based on RAPD and ISSR pattern results revealed that these isolates were not a region or host-specific (Fig. 4), which indicated the distribution of *Trichoderma* isolates in wide agroclimatic zones and also its association with multiple host systems.

**Table 2:** Summary of genetic diversity obtained by ISSR primers.

Sl. no	Primer name	Primer sequence (5′–3′)	Tm value of primer (°C)	Polymorphic bands/total no of bands	PIC*
1	ISSR02	CTCTCTCTCTCTCTCTAC	40	2/5	0.764
2	ISSR03	CTCTCTCTCTCTCTCTGC	40	1/4	0.743
3	ISSR04	CACACACACACAAC	45	3/7	0.874
4	ISSR05	CACACACACACAGT	45	3/4	0.746
5	ISSR06	CACACACACACAAG	45	1/2	0.453
6	ISSR07	CACACACACACAGC	45	1/3	0.650
7	ISSR10	GAGAGAGAGAGACC	48	2/5	0.756
8	ISSR12	CACCACCACGC	32	1/3	0.556
9	ISSR13	GAGGAGGAGGC	32	1/4	0.759
10	ISSR14	CTCCTCCTCGC	45	4/6	0.834
11	ISSR15	GTGGTGGTGGC	32	1/4	0.460
12	ISSR16	GAGAGAGAGAGAGAGAGAT	48	3/7	0.854
	Average			23/54	0.704

*PIC* polymorphic information content.

**Figure 5:**
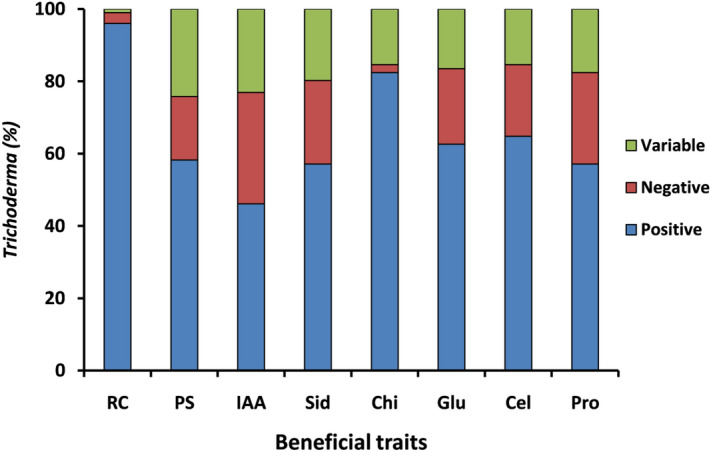
Beneficial traits of *Trichoderma* isolated across different agroclimatic zons of India. *RC* root colonization, *PS* phosphate solubilization, *IAA* indole acetic acid production, *sid* siderophore production, *Chi* chitinase, *Cel* cellulase, *Pro* protease activity.

All the 91 *Trichoderma* isolates were screened for their beneficial traits. In the root colonization study, 100% positive results were noticed in all *Trichoderma* seed treatment under greenhouse conditions (Supplementary Fig. S4). In phosphate solubilization assay, 58.2% of *Trichoderma* isolates showed a strong ability to solubilize phosphate complex present in the culture media. In contrast, only 46.1% of isolates were found producing IAA. Among the 91 isolates tested for the production of lytic enzymes, 57.2% isolates were found positive for siderophore production, 82.4, 62.6, 64.8, and 57.1% isolates were found positive for chitinase, glucanase, cellulase, and protease activity, respectively. But, in all the above studies, a certain number of *Trichoderma* isolates recorded variable results (Fig. 5).

**Figure 6:**
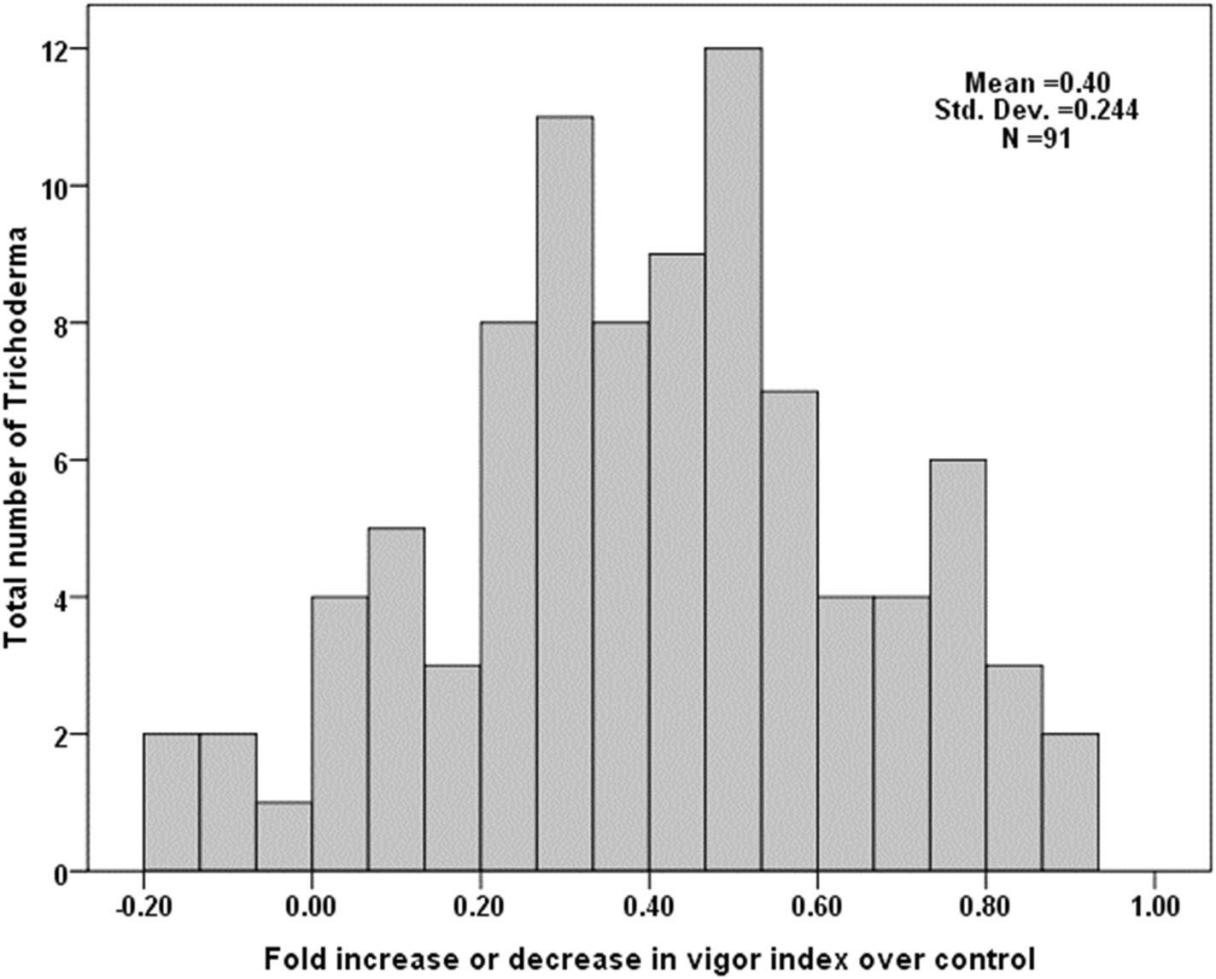
Fold changes in vigor index of pearl millet seedlings over control upon seed treatment with different *Trichoderma* spp.

All the 91 *Trichoderma* spp. were screened for their ability to improve seed quality parameters of PM. PM seedlings were analyzed for fold increase or decrease in vigor index over control upon seed treatment with different *Trichoderma* spp. Among the 91 *Trichoderma* isolates, 53 isolates showed significantly (*p* ≤ 0.05) highest seedling vigor index. None of the treatments inhibited the normal growth of seedlings. It was recorded that the seedling vigor of primed seeds was significantly (*p* ≤ 0.05) higher on an average of four-fold increase over control. Seed treatment with Metalaxyl provided 88% germination of seeds and 1498 seedling vigor, which were not significantly (*p* ≤ 0.05) different from the control (Fig. 6).

**Figure 7:**
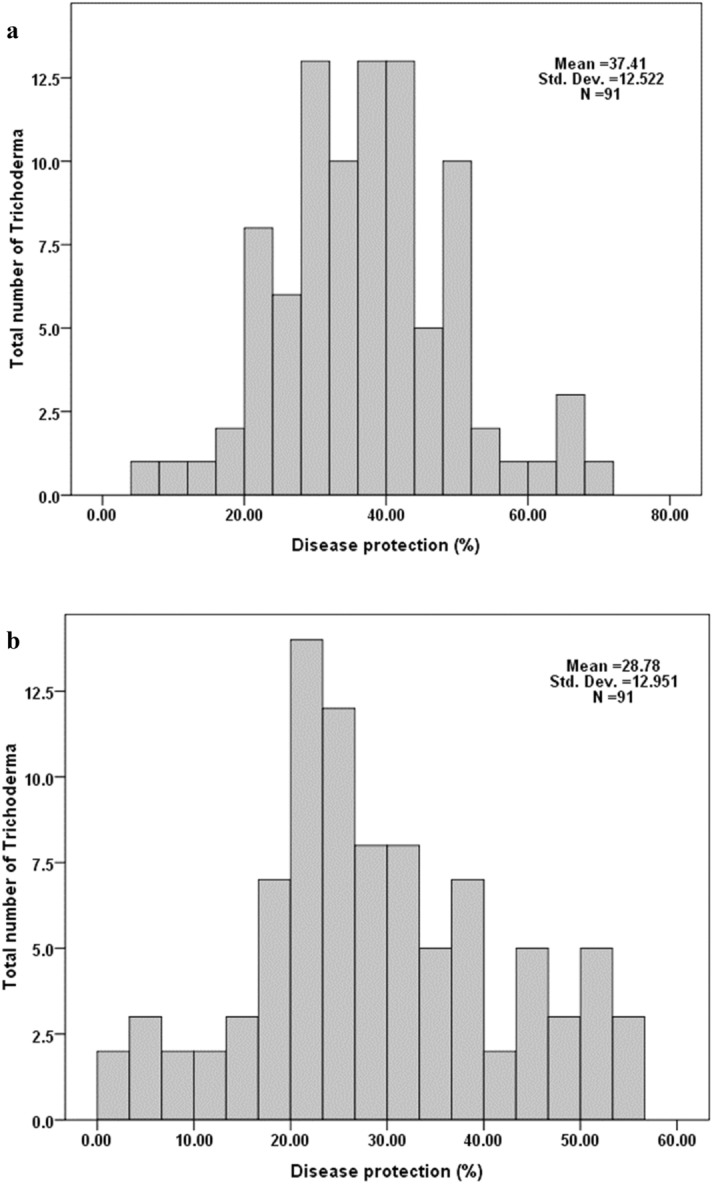
Histogram representing distribution of disease protection pattern among *Trichoderma* when employed as seed treatment. (**a**) Pathogen inoculated as soil amendment and (**b**) Pathogen inoculated as soil amendment followed by whorl inoculation.

It was recorded that the disease protection ability of *Trichoderma* spp. was significantly (*p* ≤ 0.05) higher (mean 37.41) when the pathogen was inoculated only as soil inoculation. However, the combined application of pathogen (soil and whorl inoculation) significantly (*p* ≤ 0.05) decreased the disease protecting ability of *Trichoderma* (mean 28.78). Control treatment with sterile distilled water (SDW) showed maximum disease incidence of 95.6% (soil treatment) and 93.8% (soil treatment + whorl inoculation) (Fig. 7).

**Figure 8:**
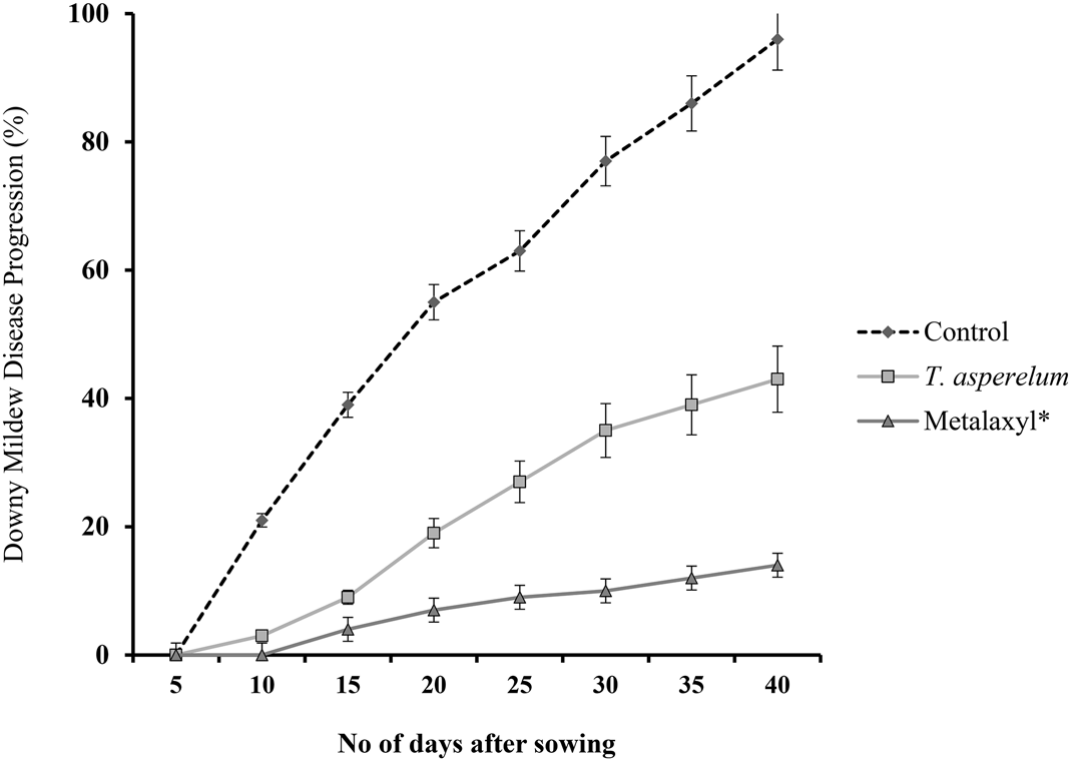
Effect of *T. asperellum* (DL-81) treatment against downy mildew disease of pearl millet under field conditions. Values ± SE (standard error) is means of three independent replicates.

Effect of seed priming with *T. asperellum* (DL-81) formulation was evaluated in DM sick plot under epiphytotic field conditions. Maximum protection with least incidence was observed in *T. asperellum* (DL-81) treated seedlings with maximum protection with least disease incidence, but in the case of the control treated plot, the highest disease incidence i.e., 96% was noticed and had not shown any protection against DM pathogen in the host plant (Fig. 8). The seed quality parameter of *T. asperellum* treatment on growth parameters of PM plants under field conditions was summarized in Table 3. Chemical metalaxyl treatment exhibited maximum of 86% disease protection.

**Table 3:** Effect of seed treatment with *T. asperellum* (DL-81) on pearl millet growth parameters under field conditions.

Growth parameters	Control	Treatment
Plant height (cm)^$^	56.2 ± 4.25^b^	73.4 ± 5.69^a^
No. of productive ear head/plant*	2.4 ± 0.23^b^	3.4 ± 0.21^a^
Length of ear head (cm)^#^	8.1 ± 0.98^b^	10.5 ± 1.24^a^
Girth of ear head (cm)^@^	3.5 ± 0.52^b^	4.1 ± 0.68^a^
1000-seed weight (grams)^€^	7.8 ± 0.87^b^	8.9 ± 0.79^a^

Values ± SE (standard error) are means of three independent replicates; Means followed by the same letter(s) within the row are not significantly different according to Tukey’s HSD at *P* ≤ 0.05 levels.

^$^As measured from the base to the tip of the plant.

*Number of earheads produced by the main axis and the basal tillers of the plant.

^#^As measured from the base to the tip of the earhead.

^@^Measured as the circumference of the earhead at the centre.

^€^Calculated by weighing 1000 seed in five replicates.

## Discussion

In this study, we intended to understand the distribution patterns of *Trichoderma* across different agroclimatic zones, functional diversity, and about beneficial interaction with plants and possible applications to improve the growth and health of PM. Distribution of *Trichoderma* is governed by several factors which include cropping pattern^38^, root exudates^39–41^, water availability, low osmotic levels^42,43^, soil pH^44,45^, temperature^46^, metal ions^47^, pesticides^48^, other biotic and abiotic stress conditions. In spite of this, *Trichoderma* is known to adapt to variable conditions and survive by imposing its beneficial effects on the local environment. These observations are encouraging in the sense that these fungi can be used to develop bioformulations, which can be applied in multiple agroclimatic conditions and also to different plant host systems.

Though more than 200 species of *Trichoderma* were reported across the world, including India. In the present study, we have observed only nine species predominantly associated with PM, wheat, and maize. Previously, Blaszczyk et al*.*^49^ reported fourteen *Trichoderma* spp. among which, *T. harzianum* was found to be the most abundant in Poland. In South Africa, Du-Plessis et al*.*^50^ recorded fourteen different species of *Trichoderma* among which ten were for the first time reported in this region. In Manipur (India), 22 different species of *Trichoderma* were identified from nine geographically diverse zones. They were diverse in their phylogeny, and *T. harzianum* was most predominant in occurrence^51^. In the present investigation, *Trichoderma* isolates exhibited significant intra-species and inter-species genetic variations as observed through RAPD and ISSR analysis.

With evidence from the similar average values of PIC (0.718 for RAPD and 0.704 for ISSR), RAPD and ISSR based assays were found equally useful for the analysis of genetic diversity of isolated *Trichoderma* spp. Also, Mantel test reveals the weak correlation (R^2^ = 0.095) among genetic distance analyzed by RAPD and ISSR data. This indicates that these two sets of markers target different regions of the *Trichoderma* genome. Similar results of low levels of correlation between RAPD and ISSR markers were observed in microbes and higher plants^52^.

In the present study, the UPGMA cluster analysis based on RAPD and ISSR data, studied 91 *Trichoderma* isolates were not clustered according to their place of collection or host plant (Fig. 4). This may be due to the high genetic diversity and wide spread of *Trichoderma* spp. across the India. Moreover, in the UPGMA cluster, AP-42 isolate (*T. harzianum*, collected from Guntur, Andhra Pradesh, India, from the host plant PM), followed by BG-21 (*T. longibrachiatum*, collected from Bagalkote, Karnataka, India, from the host plant maize) were grouped distantly separated from the other accession, suggesting the lowest genetic identity of these accessions with others.

The PCA plot constructed from RAPD and ISSR binary matrix showed the substantial distribution of *Trichoderma* spp. collected from the different agroclimatic regions (Fig. 5). Moreover, the wide distribution of *Trichoderma* spp. in the PCA plot revealed the existence of significant genetic diversity. The presence of considerable bio-diversity of *Trichoderma* spp. from soils of the particular region offers opportunities for further sustainable agriculture management practices, especially in the progress of identifying the bio-control agent to manage plant diseases^53^.

New-fangled haplotypes have been documented in *T. harzianum* based on the study of ITS sequences phylogenetic analysis and were substantiated by data on RAPD profile^54^. Samuels et al*.*^55^ differentiated *T. aggressivum* f. *aggresivum* and *T. aggressivum* f. *europaeum* from morphologically parallel species based on temperature growth associations, examined the aptitude to cause green mold disease of mushrooms, and further analyzed the ITS-1 region, elongation factor gene sequence. Hermosa et al*.*^56^ studied the genetic diversity of 69 biocontrol isolates of *Trichoderma* spp. from different geographical regions and culture collections based on the examination of sequence data attained from the ITS1 province and a segment of the tef1 gene. Growth promoting and beneficial traits of *Trichoderma* isolates are highly correlated and found varying from isolate to isolate. It was observed that chitinase uses an essential mechanism of pathogen suppression and also IAA production and phosphate solubilization is important for plant growth improvement. These benefical traits are extensively studied and correlated with plant growth, health, biomass, and yield of the plant^57,58^. Phosphate solubilization, phytohormone production, lytic enzymes, and antibiotic production are the main characteristics of *Trichoderma* in promoting plant growth and biocontrol activity^59,60^.

In general, the mechanism involved in plant disease suppression by *Trichoderma* is identified as, directly through antagonism or indirectly by elicitation of plant-defense responses^61,62^. Disease protection by direct suppression is through mycoparasitism^63,64^ fabrication of lytic enzymes such as chitinase^65^, β-1, 3 glucanase^66,67^, protease^68^, and antimicrobial chemicals (Trichoharzianol, Isoharziandione, 6-pentyl-α-pyrone)^69–71^ and competing for space and food. Disease protection by ISR has been reported in several plant disease managements by *Trichoderma* spp. including PM-DM^72,73^.

Cell wall glucan of *T. hamatum* UOM 13 was found to suppress the DM diseases in PM by inducing systemic resistance^74^. Further Siddaiah et al*.*^73^ reported that the UOM 13 induced resistance was through enhanced structural defense (lignification and callose deposition), upregulation of defense-related enzymes (Glucanase, Peroxidase, Phenylalanine ammonia-lyase, Polyphenol oxidase), PR protein, and Hydroxyproline-Rich Glycoproteins in challenged PM seedlings. Involvement of β-1,3-glucanase in suppressing the DM disease was demonstrated by O'Kennedy et al*.*^75^ were the genes of β-1,3-glucanase (gluc78) from *T. atroviride*, was introduced into the genome of pearl millet (842B), by particle bombardment and expressed. Further, a clear correlation was established between increased β-1,3-glucanase activity and decreased DM disease in transgenic PM seedlings. In our earlier studies^18,76^ the capabilities of *Trichoderma* crude protein and lipid fractions of *T. brevicompactum* (UP-91) to induce systemic resistance against DM infection was observed. Further, the active lipid molecule involved in ISR is suspected as (E)-N-(1,3-dihydroxyoctadec-4-en-2-yl) acetamide. The colonization of the rhizosphere by *Trichoderma* spp. produces direct positive effects on plants, promoting their growth, and activating their defense mechanisms. *T. asperellum* and *T. atroviride* modulated salicylic acid, jasmonic acid, and antifungal defensin pathways in tomato plants during the early stage of the root pathogen infection^77^. *T. harzianum* ETS323 secrets an flavoenzyme, L-amino acid oxidase has antimicrobial characteristics by activating H_2_O_2_ signaling defense mechanisms to confer resistance against *S. sclerotiorum* and *B. cinerea* in tobacco^78^.

We have studied *S. graminicola,* an oomycetous pathogen in PM host plant, and its influence and modulation on biochemical parameters upon seed priming with *Trichoderma* spp. As the major cell wall constituents of oomycetes are β-1, 3-glucan^79^, it is believed that glucanase will play a crucial role in suppressing DM pathogen over chitinase. In the present study, most of the *Trichoderma* isolates recorded significant (*p* ≤ 0.05) reduction in DM incidence, when the pathogen was inoculated in the soil. Though, the detailed disease protection mechanisms were not analyzed in the current study. It was presumed that it follows various mechanisms mentioned above. However, their disease protection ability was found to decrease after providing an additional whorl inoculation of the pathogen. This observation revealed that the principal mechanism with which *Trichoderma* reduces the disease is by suppressing pathogen by directing inhibition. However, their ability to ISR varies among isolates. The present study showed the current status of *Trichoderma* distribution in different PM growing agroclimatic regions of India. Moreover, the wide occurrence of nine *Trichoderma* spp. beyond the barriers of environmental conditions and the host system enhanced the probability of employing these bioagents for the improvement of PM growth and health.

## Methods

### Isolation, identification, and characterization of *Trichoderma* spp

To isolate *Trichoderma* spp., field surveys were conducted under different agroclimatic zones of India, which includes 10 different states (Karnataka, Tamil Nadu, Andhra Pradesh, Madhya Pradesh, Maharashtra, Rajasthan, Gujarat, Haryana, Uttar Pradesh, and Delhi) belonging to eight different agroclimatic zones (Supplementary Fig. S1), in between September 2012 to January 2014. In each field, rhizosphere soils of five randomly selected healthy looking plants were collected and pooled to obtain a composite sample. Soil samples were collected into sterile cotton bags, transported to the laboratory, and immediately processed.

Rhizospheric soil (10 g) was mixed with 250 ml of phosphate-buffered saline containing streptomycin (100 mg/l) and penicillin (100 mg/l) to suppress the native bacterial growth and incubated at 28 ± 2 °C for 2 h on a rotary shaker at a speed of 150 rpm. Ten-fold serial dilutions were prepared and from each dilution, 100 μl were inoculated by spread plate method on Martin medium^80^ amended with streptomycin (100 mg/l) and penicillin (100 mg/l). The inoculated plates were incubated for 7 days at 28 ± 2 °C and visually observed for the fungal growth at regular time intervals. At the end of the incubation period, the suspected fungus was isolated onto potato dextrose agar (PDA) slants.

The fungal isolates were identified based on their morphological nature by growing them on PDA at 28 ± 2 °C for 5–7 days. The fungal colonies were visually observed for their color (obverse and reverse), texture, margin, and sporulation. Individual fungal isolates were identified at the species level using morphological keys and species descriptions (conidiophores, shape, size, arrangement, and phialides arrangements were analyzed microscopically) as per the protocol suggested by Rifai^81^ and Leahy et al*.*^82^. Fungal strains were sub-cultured and grown on PDA for routine experiments and the long-term storage, fungal slants were prepared in cryovials overlaid with 20% glycerol and stored at − 80 °C.

Genomic DNA was extracted following the method of Saghai-Maroof et al*.*^83^. PCR amplification of ITS region was performed using forward ITS1 (5′ TCC-GTA-GGT-GAA-CCT-GCG-G 3′) and reverse ITS4 (5′ TCC-TCC-GCT-TAT-TGA-TAT-GC 3′) primers following the conditions given by White et al*.*^84^. Further, the terminal regions of DNA sequence with deprived alignment were manually detached via BioEdit v 7.0.5.3 and subjected to BLAST searches to consign putative identity, the depiction of operational taxonomic entities based on phylogenetic implication and sequence similarity measures. The sequences were deposited in the NCBI database and an accession number was obtained (Supplementary Table S1).

Molecular Phylogenetic analysis and evolutionary history were grouped based on the Tamura-Nei model by using the Maximum Likelihood method^85^. The bootstrap consensus tree was inferred from 1000 replicates taken to symbolize the evolutionary history of the taxa examined using MEGA6^86,87^.

### Genetic diversity analysis

A set of 20 RAPD primers and 12 ISSR primers were used for the polymorphism study (Table 2). RAPD analysis was performed in 0.2 ml PCR vials including reaction buffer, 1 µl of dNTP mix (2.5 M each), 2.5 µl of 10 × PCR buffer, 1U Taq DNA polymerase, 30 ng DNA template, 10 pmol of 1 µl primer, and DNase free water to a final volume of 25 µl. The amplification of the PCR reaction was carried out at 94 °C for 3 min (initial denaturation), followed by 45 cycles of 94 °C for 1 min (denaturation); 40 °C for 1 min (annealing temperature of primer); 72 °C for 2 min (extension of primer) and 72 °C for 10 min (final extension).

For the ISSR analysis, annealing temperature differs for the specific primer based on Tm (Table 3), and other conditions were the same as explained for RAPD. PCR amplified products were separated and analyzed on the agarose gel. The sizes of the amplicon were calculated with a DNA ladder of 100–5000 bp and imaged using a gel documentation system (BioRad, Canada). For each DNA sample, the process of RAPD and ISSR analysis was performed twice and primers with reproducible nature were subjected to diversity analysis.

The amplified products of different sizes were scored as 1 (presence) and 0 (absence) for 91 *Trichoderma* spp. to produce a binary matrix. Analysis of data was carried out using the software NTSYS-pc, version 2.11w (Numerical Taxonomy System Biostatistics) to analyze the Jaccard’s similarity coefficients^88^. The SIMQUAL program was employed to estimate the Jaccard’s coefficients. The dendrogram was produced by grouping Jaccard coefficients using the Sequential agglomerative hierarchical non-overlapping (SAHN) clustering program, choosing the algorithm of Unweighted Pair-Group Methods with Arithmetic Average (UPGMA) in NTSYS-pc^89^.

Polymorphic information content (PIC) was studied using the method: PIC = 1 − ∑pi2, where ‘pi’ is the frequency of the *i*th allele^90^. Percent polymorphism was calculated according to the principle: % Polymorphism = p/(m + p), where p is a sum of the number of polymorphic bands and m is the total number of monomorphic bands of the primer alliance used. Multiplex ratio (MR) for all markers was determined via the formula: MR = (m + p)/n, where p is the total number of polymorphic bands, m is the total number of monomorphic bands and n is the total number of primer combinations^91^. Average heterozygosity (Hav) was known by taking the average of PIC values acquired for all the markers. Marker index (MI) was studied by multiplying the Hav with the MR. Genetic diversity of the 91 *Trichoderma* spp. and its interpretation was performed by the method explained by Saini et al*.*^52^.

### Characterization of *Trichoderma* spp. for their beneficial traits

#### Root colonization bioassay

Seeds of PM (cv. 7042S) which was reported as highly susceptible to DM pathogen were obtained from the International Crop Research Institute for Semi-Arid Tropics, Patencheru, India. Sodium hypochlorite (0.2%) was used for the surface sterilization of seeds, later thoroughly washed with SDW, and used throughout the experimental studies. Pure cultures *Trichoderma* spp. were obtained by growing on PDA. 10 ml SDW was added to PDA plates and conidia were dislodged from the culture surface and the concentration was adjusted to 1 × 10^8^ conidia/ml using a hemocytometer.

PM seeds were treated with a conidial suspension of *Trichoderma* spp. amended with 0.2% Carboxymethylcellulose (CMC) as adhesive for 3 h at 28 ± 2 °C on a rotary shaker (150 rpm). Seeds primed only with SDW amended with CMC were referred to as control. Treated seeds were sown thickly in pots (22 cm diameter) filled with the sterilized potting mixture [soil: farmyard manure: sand (2:1:1, v/v/v)]. The earthen pots with different treatments were randomly arranged in block designs and maintained under greenhouse conditions at 25–30 °C with 95% relative humidity (day-night light cycle consisting of an average temperature and light of 18 ± 2 °C in the night and 28 ± 2 °C in the day with natural light). After 7 days, the seedlings were thinned to space out by leaving six to eight seedlings per pot. After 21 days of incubation, plants were carefully removed without damaging the roots and the rhizosphere soil was collected. The soil was processed as explained above and the colony-forming unit (cfu) was determined following the serial dilution technique on Martin’s media. Visual and microscopic observations were carried out as explained earlier. For further confirmation, root bits (3 mm) were placed on Martin media, and the fungal growth was analyzed. All bioassays were performed in triplicates of six pots each and repeated twice.

#### Indole acetic acid production

*Trichoderma* spp. were grown in potato dextrose broth (PDB) amended with L-Tryptophan (0.1 g/L) for 4–5 days at 28 ± 2 °C on a rotary shaker at 150 rpm. At the end of the incubation period, mycelia and debris were separated by filtration and centrifugation, respectively. To 1 ml of the culture filtrate, 4 ml of Salkowski reagent (2% 0.5 M ferric chloride in 35% perchloric acid) was added, and development of pink to red color was visually observed after 10 min of the incubation period in dark condition^92^. An uninoculated PDB was maintained as a control. For each isolate, the experiment was conducted in triplicates and repeated thrice.

#### Phosphate solubilization

Calcium phosphate solubilization ability of different *Trichoderma* spp. was determined by growing them on National Botanical Research Institute’s Phosphate growth medium (NBRIP)^93^. The petriplates containing NBRIP was point-inoculated and incubated at 28 ± 2 °C for 4–5 days. Change in color of the media surrounding or beneath the fungal media from purple to yellow indicates the ability of an isolate to solubilize calcium phosphate.

#### Siderophore production

*Trichoderma* isolates were point inoculated onto Chrome azurol S (CAS) media and incubated for 5–7 days at 28 ± 2°C^94^. Development of pink or yellow or orange coloration around or below the fungal colony indicates the positive nature of fungal isolate to produce siderophore.

### Lytic enzyme assay

The *Trichoderma* isolates were grown on 1/10th strength PDB for 48 h on a rotary shaker at 150 rpm at 28 ± 2 °C. For induction of enzyme production, the specific substrate (CMC for cellulase; colloidal chitin for chitinase; laminarin for β, 1–3, glucanase and casein for protease) was added at 0.5% (w/v) into the culture medium. Further, the incubation was continued for 24 h, and culture filtrate was separated by filtration and centrifugation (7800*g*; 4 °C; 10 min) and used as an enzyme source.

Chitinase (EC 3.2.1.14) activity was quantified spectrophotometrically. Briefly, 100 µl of the culture filtrate was blended with 200 µl of colloidal chitin (0.5% in 50 mM acetate buffer pH 5.2). The reaction was incubated for 60 min at 40 °C with intermediate shaking. To stop the reaction, the vials were placed in a boiling water bath for 5 min. After cooling, 3 ml of freshly prepared dimethylaminobenzaldehyde solution was added and incubated at 40 °C for 60 min. A standard graph was prepared using different concentrations of N-Acetylglucosamine (GLcNAc) and the enzyme activity was measured by quantifying the released GLcNAc^95^.

β-1,3 glucanase (EC 3.2.1.39) activity was evaluated by adding100 µl culture supernatant to 200 µl of laminarin (5%, w/v) prepared in 50 mM acetate buffer (pH 5) and further incubated at 40 °C for 10 min. Reducing sugar was measured by the 3,5-dinitrosalicylic acid (DNS) method^96^.

Cellulase activity (EC 3.2.1.21) was examined by adding 100 µl of the culture supernatant to 400 µl of CMC (1%) prepared in 100 mM sodium citrate buffer (pH 5.2) and the assay mixture was further incubated at 55ºC for 15 min. The reducing sugars released were determined by the DNS technique^96^.

To analyze Protease (EC 3.4.21.4) activity, 500 µl of the culture supernatant was added to 500 µl of casein (0.36%) and 2 ml of acetate buffer (100 mM, pH 3.6). Towards the end of the reaction period (1 h, 50ºC), 3 ml of trichloroacetic acid (5%) was added to stop the reaction. Blank was considered a zero period of incubation. Free amino acids released were measured by the Ninhydrin method^97^.

The concentration of proteins in the samples were verified using the Bradford method^98^, with bovine serum albumin (BSA; Sigma Aldrich, St. Louis, USA) as a standard. All enzyme assays were replicated three times for each sample.

The specific activity was articulated as Unit mg^−1^ protein and Unit mg^−1^ aminoacid (for protease). The activity is described as the quantity of enzyme required to generate one mM of equivalent reducing sugar in one min of one ml culture supernatants. Non-enzymatic controls were also carried out using boiled enzymes and were deducted from the enzymatic values.

### Effect of *Trichoderma* seed treatment on seed quality variables of pearl millet

Seed treatment was conducted aseptically as explained earlier in the laminar airflow and the seeds were dried overnight and used. Seed germination was tested following the paper towel method^99^. Mean root length (MRL) and Mean shoot length (MSL) were measured and seedling vigor was calculated using the following formula^100^.

$$ {\text{Vigor index }} = \, \left( {{\text{MRL}} + {\text{MSL}}} \right) \, \times \, \% {\text{ Germination}} $$

For each treatment, four samples of 100 seeds were used, and the experiment was replicated thrice.

### Disease protection studies

*Sclerospora graminicola* sick plot is being maintained in the University of Mysore at Department of Biotechnology, Mysuru, for the last 30 years under the Indian Council of Agricultural Research (ICAR)-All India Coordinated Pearl Millet Improvement Project (AICPMIP, Jodhpur Rajasthan, India). The pathogen was isolated from the highly susceptible PM leaves grown in the sick plot. Infected leaves were cleaned with running tap water to eliminate debris and existing sporangia, blot-dried, and incubated in a moist chamber at 80% humidity at 20 °C under dark conditions. Fresh sporangia formed on the infected leaves were harvested in SDW, and spore concentration was adjusted to 4 × 10^4^ per ml using a hemocytometer and utilized as inoculum^101^. Oospores were obtained from the infected leaves of pearl millet which was containing approximately 5 × 10^5^ oospores per gram dry weight.

Greenhouse conditions and the methods of the pot experiment were performed as explained earlier. Disease protection studies were conducted in two sets of experiments, (1) control and treated seeds were raised in sterile soil and pathogen (zoospores) was challenge inoculated as whorl inoculation^102^ and (2) control and treated seeds were raised in soil containing pathogen (~ 5 × 10^5^ oospores) and an additional whorl inoculation was performed as explained above. In both cases, the challenge-inoculated plants were maintained under greenhouse conditions (80% RH). Disease frequency was monitored by examining the number of plants that confirmed any one of the characteristic DM symptoms (chlorosis, sporulation on the abaxial leaf surface, stunted growth, etc.) up to 30 days after sowing. For each *Trichoderma* isolate, eight pots of two replicates were maintained, and the experiment was repeated twice.

*Trichoderma* treatment offering the best protection under greenhouse conditions against DM disease was selected for the field trials. Field studies were conducted in the DM sick plot of DOS in Biotechnology, University of Mysore (N24° 18′, E79° 26′, 903 m altitude, red loam soil). This experimental station has been maintained over three decades under the ICAR program, with a severe infestation of the DM oospores inoculum and additional inoculum in the form of asexual spores is provided from spreader rows raised 21 days before sowing of experimental plot^103^. The treated seeds were sown in randomized block design 10 × 6 m plots with four replications per treatment. Each row was 6 m long and 75 cm apart with 15 cm spacing between plants within rows. Susceptible controls were similar as described earlier. The plants were raised following recommended agronomical practices. Disease incidence was recorded at 30 and 60 days growth of seedlings, as the plants started showing typical DM disease symptoms.

### Plant growth promotion and yield analysis under field conditions

Seed treatment with *T. asperellum* (DL-81) and seedlings were raised in the field as explained above. Except, these set of experiments were conducted in DM disease-free plots and the seedlings were not subjected to challenge inoculation with pathogen. Towards the end of the growth period (60 days), plant height (cm), Number of productive ear head, Length of ear head (cm), Girth of ear head (cm), and 1000-seed weight (g) was measured and tabulated.

### Statistical analysis

All the data from laboratory and greenhouse experiments were subjected to one-way analysis of variance (ANOVA) using SPSS, version 17. The significant difference between the averages of treatments were compared using Highest Significant Difference (HSD) as attained by Tukey test at *p* ≤ 0.05 levels.

### Ethical approval

All authors concur with the submission and have seen a draft copy of the manuscript and agree with its publication. The manuscript does not contain experiments using animals and human studies.

## Supplementary Information

Supplementary Information
