# Supplementary material for: Trichovariability in rhizosphere soil samples and their biocontrol potential against downy mildew pathogen in pearl millet
Source: Sci Rep. 2021 May 4;11:9517. doi: 10.1038/s41598-021-89061-2 (PMC8096818; doi:10.1038/s41598-021-89061-2)
Supplement: Supplementary file 1 — Supplementary Information [file 41598_2021_89061_MOESM1_ESM.pdf]

Scientific Reports

Supplementary Information

Trichovariability in rhizosphere soil samples and their biocontrol potential against downy mildew pathogen in pearl millet

Boregowda Nandini [1], Hariprasad Puttaswamy [2], Ramesh Kumar Saini [1, 3], Harischandra Sripathy Prakash[1] & Nagaraja Geetha [1]*

Affiliations

1. Department of Studies in Biotechnology, University of Mysore, Manasagangotri, Mysuru, Karnataka, India.
2. Centre for Rural Development and Technology, Indian Institute of Technology Delhi, New Delhi, India
3. Department of Crop Science, Konkuk University, Seoul 143-701, Republic of Korea

*** Correspondence author:**

Dr. Geetha. N,
Assistant Professor,
Department of Studies in Biotechnology,
University of Mysore, Manasagangotri,
Mysuru-570 006,
Karnataka, India.

Email id: geetha@appbot.uni-mysore.ac.in; dignathjogi1@gmail.com

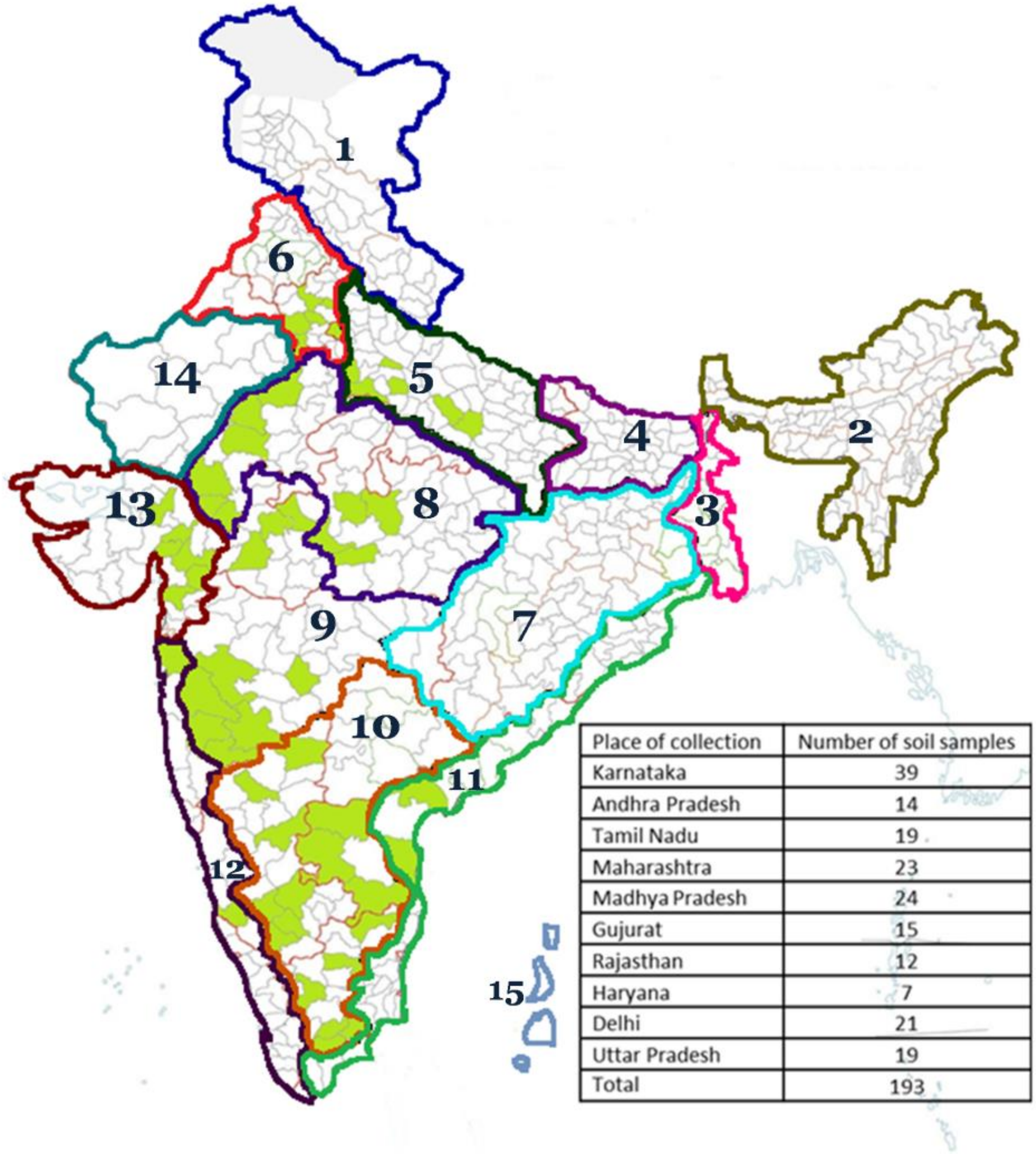

Place of collection	Number of soil samples
Karnataka	39
Andhra Pradesh	14
Tamil Nadu	19
Maharashtra	23
Madhya Pradesh	24
Gujurat	15
Rajasthan	12
Haryana	7
Delhi	21
Uttar Pradesh	19
Total	193

S. Figure 1. Field survey for the collection of rhizosphere soil samples from different pearl millet growing zones of India. Agroclimatic zones : 1. Western Himalayan division, 2. Eastern Himalayan division, 3. Lower Gangetic plain region, 4. Middle Gangetic plain region, 5. Upper Gangetic plain region, 6. Trans-Gangetic plain region, 7. Eastern plateau and hill region, 8. Central plateau and hill region, 9. Western plateau and hill region, 10. Southern plateau and hill region, 11.East coast plain and hill region, 12.West coast plain and hill region, 13.Gujarat plain and hill region, 14.Western plain and hill region, and 15. Island region.

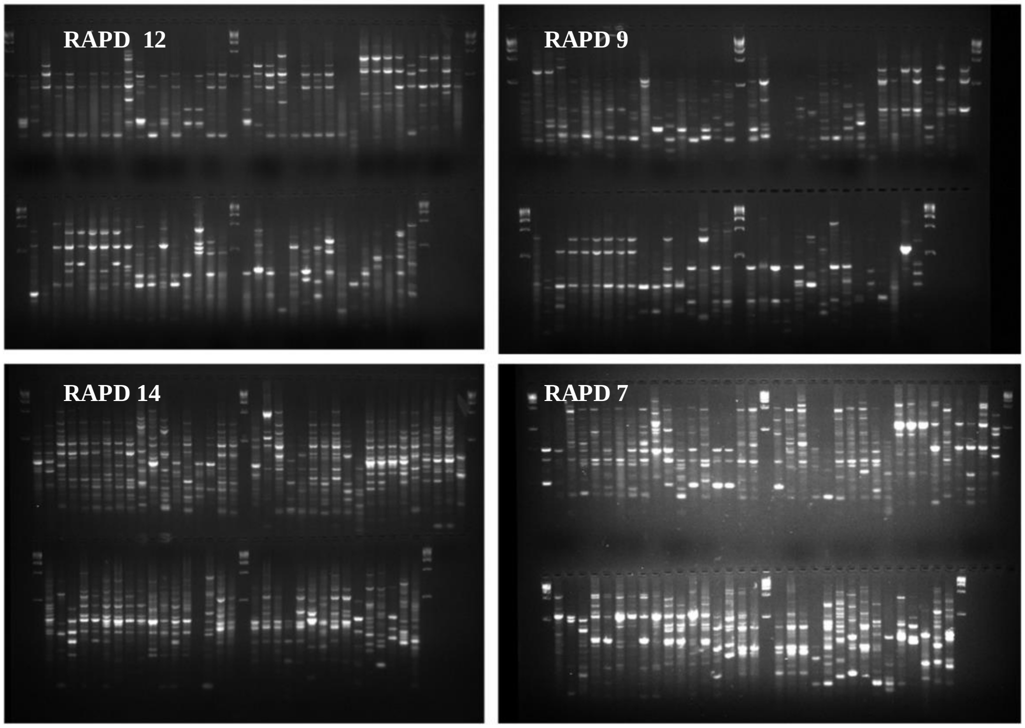

S. Figure 2. An illustration of representative RAPD analysis of 91 *Trichoderma* spp.

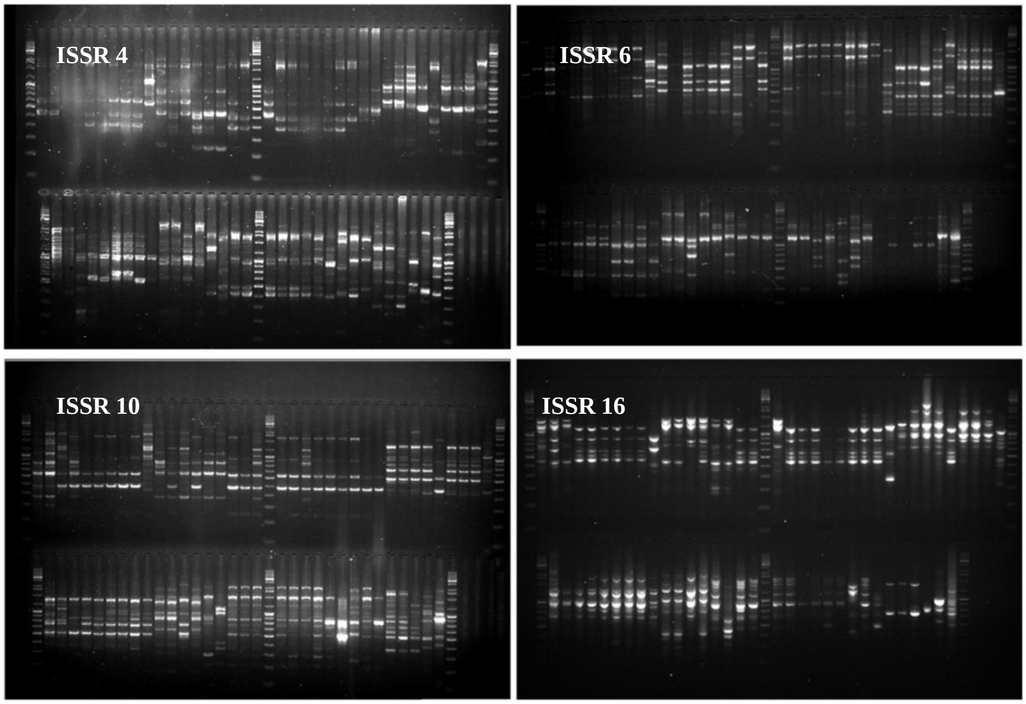

S. Figure 3. An illustration of representative ISSR analysis of 91 *Trichoderma* spp.

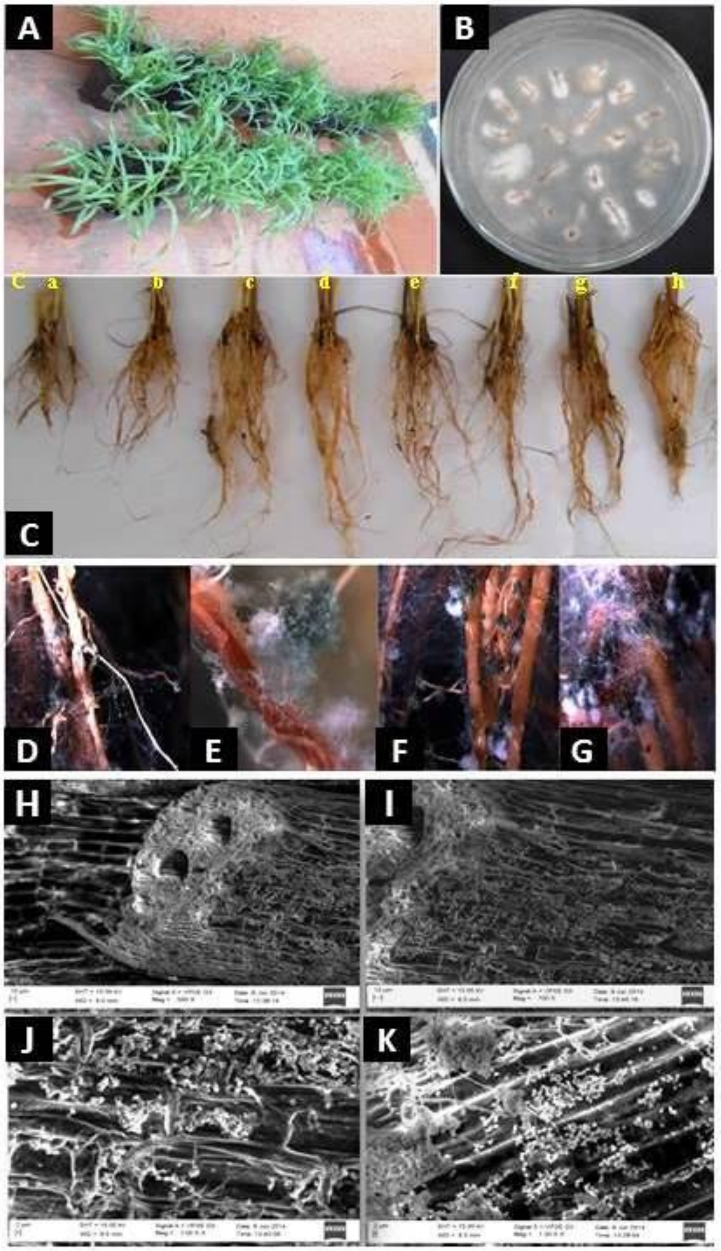

S. Figure 4. Root colonization studies

A: Seedlings grown under green house conditions for root colonization assay by treating with *Trichoderma* conidial suspension, B: Growth of the mycelium of *Trichoderma* spp. on root bits on PDA plate, C: Root length of plants treated with different *Trichoderma* spp. compared with control [a - Distilled water (Control); b to h – different *Trichoderma* treatment], D, E, F, G: root colonization observed under stereomicroscope, H, I, J and K: Scanning Electron Microscopic view of colonization of *Trichoderma* on the surface of root.

Supplementary Table 1. Details of the *Trichoderma* spp. isolated from different agroclimatic zones of India used in the present study.

Agro-climatic zone	Isolate Code	Identity	Accession number	Place of Collection	Host plant
10	MY-1	*T. asperellum*	KM043483	Mysuru, Karnataka, India	Pearl millet
10	MY-2	*T. harzianum*	KM043484	Mysuru, Karnataka, India	Pearl millet
10	MY-3	*T. harzianum*	KM043485	Mysuru, Karnataka, India	Pearl millet
10	MY-4	*T. asperellum*	KM043486	Mysuru, Karnataka, India	Pearl millet
10	MY-5	*T. asperellum*	KM043487	Mysuru, Karnataka, India	Pearl millet
10	MY-6	*T. virens*	KM043488	Mysuru, Karnataka, India	Pearl millet
10	MY-7	*T. asperellum*	KM043489	Mysuru, Karnataka, India	Pearl millet
10	MY-8	*T. harzianum*	KM043490	Mysuru, Karnataka, India	Pearl millet
10	MY-9	*T. longibrachiatum*	KM043491	Mysuru, Karnataka, India	Pearl millet
10	MD-10	*T. harzianum*	KM100756	Mandya, Karnataka, India	Maize
10	MD-11	*T. harzianum*	KM100757	Mandya, Karnataka, India	Maize
10	MD-12	*T. harzianum*	KM100758	Mandya, Karnataka, India	Maize
10	MD-13	*T. virens*	KM100759	Mandya, Karnataka, India	Maize
10	MD-14	*T. virens*	KM100760	Mandya, Karnataka, India	Maize
10	MD-15	*T. harzianum*	KM100761	Mandya, Karnataka, India	Maize
10	MD-16	*T. virens*	KM100762	Mandya, Karnataka, India	Maize
10	MD-17	*T. asperellum*	KM100763	Mandya, Karnataka, India	Maize
10	MD-18	*T. asperellum*	KM100764	Mandya, Karnataka, India	Maize
10	MD-19	*T. asperellum*	KM100765	Mandya, Karnataka, India	Maize
10	BG-20	*T. longibrachiatum*	KM100787	Bagalkote, Karnataka, India	Bajra
10	BG-21	*T. longibrachiatum*	KM100788	Bagalkote, Karnataka, India	Maize
10	BG-22	*T. harzianum*	KM100789	Bagalkote, Karnataka, India	Maize
10	BG-23	*T. harzianum*	KM100790	Bagalkote, Karnataka, India	Maize
10	BG-24	*T. longibrachiatum*	KM100791	Bagalkote, Karnataka, India	Maize
10	BG-25	*T. harzianum*	KM100792	Bagalkote, Karnataka, India	Maize
10	BG-26	*T. atroviride*	KM100793	Bagalkote, Karnataka, India	Maize
10	GB-27	*T. harzianum*	KM100766	Gulbarga, Karnataka, India	Pearl millet
10	GB-28	*T. harzianum*	KM100767	Gulbarga, Karnataka, India	Pearl millet

10	GB-29	*T. asperellum*	KM100768	Gulbarga, Karnataka, India	Pearl millet
10	GB-30	*T. harzianum*	KM100769	Gulbarga, Karnataka, India	Pearl millet
10	GB-31	*T. virens*	KM100770	Gulbarga, Karnataka, India	Pearl millet
10	GB-32	*T. asperellum*	KM100771	Gulbarga, Karnataka, India	Pearl millet
10	GB-33	*T. asperellum*	KM100772	Gulbarga, Karnataka, India	Maize
10	GB-34	*T. asperellum*	KM100773	Gulbarga, Karnataka, India	Maize
10	GB-35	*T.asperellum*	KM100774	Gulbarga, Karnataka, India	Pearl millet
10	AP-36	*T. asperellum*	KM100775	Chittor, Andhra Pradesh, India	Maize
10	AP-37	*T. harzianum*	KM100776	Nellore, Andhra Pradesh, India	Maize
10	AP-38	*T. harzianum*	KM100777	Guntur, Andhra Pradesh, India	Maize
10	AP-39	*T. asperellum*	KM100778	Anantapur, Andhra Pradesh, India	Maize
11	AP-40	*T. asperellum*	KM100779	Anantapur, Andhra Pradesh, India	Sorghum
11	AP-41	*T. asperellum*	KM100780	Kurnool, Andhra Pradesh, India	Pearl millet
11	AP-42	*T. harzianum*	KM100781	Guntur, Andhra Pradesh, India	Pearl millet
10	TN-43	*T. virens*	KM100782	Vellore, Tamil nadu, India	Maize
10	TN-44	*T. virens*	KM100783	Virudunagar, Tamil nadu, India	Maize
10	TN-45	*T. virens*	KM100784	Coimbatore,Tamilnadu, India	Maize
10	TN-46	*T. asperellum*	KM100785	Virudunagar, Tamil nadu, India	Pearl millet
10	TN-47	*T. asperellum*	KM100786	Sivaganga, Tamil nadu, India	Pearl millet
9	MH-48	*T. asperellum*	KM100824	Solapur, Maharashtra, India	Pearl millet
9	MH-49	*T. asperellum*	KM100825	Satara, Maharashtra, India	Pearl millet
9	MH-50	*T. atroviride*	KM100830	Satara, Maharashtra, India	Pearl millet
9	MH-51	*T. asperellum*	KM100826	Solapur, Maharashtra, India	Pearl millet
9	MH-52	*T. asperellum*	KM100827	Sangli, Maharashtra, India	Pearl millet
12	MH-53	*T. asperellum*	KM100828	Ahmednagar, Maharashtra, India	Pearl millet
12	MH-54	*T. asperellum*	KM100829	Thane, Maharashtra, India	Pearl millet
8	MP-55	*T. asperellum*	KM100794	Shajapur, Madhya Pradesh, India	Pearl millet
8	MP-56	*T. asperellum*	KM100795	Shajapur, Madhya Pradesh, India	Pearl millet
8	MP-57	*T. asperellum*	KM100796	Hoshangabad, Madhya Pradesh, India	Wheat
8	MP-58	*T. asperellum*	KM100797	Ujjain, Madhya Pradesh, India	Sorghum
9	MP-59	*T. longibrachiatum*	KM100831	Hoshangabad, Madhya Pradesh, India	Pearl millet
9	MP-60	*T. virens*	KM100832	Ujjain, Madhya Pradesh, India	Pearl millet
13	GU-61	*T. virens*	KM100798	Nadiad, Gujurat, India	Pearl millet
13	GU-62	*T. harzianum*	KM100799	Mahesana, Gujurat, India	Pearl millet

13	GU-63	*T. asperellum*	KM100800	Vododara, Gujurat, India	Pearl millet
13	GU-64	*T. virens*	KM100801	Surat, Gujurat, India	Pearl millet
13	GU-65	*T. harzianum*	KM100802	Surat, Gujurat, India	Pearl millet
13	GU-66	*T. harzianum*	KM100803	Rajpipla, Gujurat, India	Pearl millet
8	RJ-67	*T. harzianum*	KM100804	Udaipur, Rajasthan, India	Pearl millet
8	RJ-68	*T. asperellum*	KM100805	Udaipur, Rajasthan, India	Pearl millet
8	RJ-69	*T. asperellum*	KM100833	Jaipur, Rajasthan, India	Pearl millet
8	RJ-70	*T. virens*	KM100806	Udaipur, Rajasthan, India	Pearl millet
8	RJ-71	*T. harzianum*	KM100807	Bhilwara, Rajasthan, India	Pearl millet
8	RJ-72	*T. harzianum*	KM100808	Jaipur, Rajasthan, India	Pearl millet
6	HR-73	*T. harzianum*	KM100834	Mahendragarh, Haryana, India	Pearl millet
6	HR-74	*T. harzianum*	KM100809	Mahendragarh, Haryana, India	Pearl millet
6	HR-75	*T. harzianum*	KM100810	Mahendragarh,Haryana, India	Pearl millet
6	HR-76	*T.* harzianum	KM100811	Rohtak, Haryana, India	Sorghum
6	HR-77	*T. harzianum*	KM100812	Jind, Haryana, India	Pearl millet
6	DL-78	*T. harzianum*	KM100813	Jind, Haryana, India	Maize
6	DL-79	*T.harzianum*	KM100814	Narela,Delhi, India	Pearl millet
6	DL-80	*T. harzianum*	KM100815	Narela,Delhi, India	Wheat
6	DL-81	*T. asperellum*	KM100835	Narela,Delhi, India	Pearl millet
6	DL-82	*T. asperellum*	KM100816	Narela,Delhi, India	Pearl millet
6	DL-83	*T. asperellum*	KM100817	Najafgarh, Delhi, India	Pearl millet
6	DL-84	*T. harzianum*	KM100818	Najafgarh,Delhi, India	Wheat
6	DL-85	*T. harzianum*	KM100819	Najafgarh,Delhi, India	Pearl millet
6	DL-86	*T. asperellum*	KM100820	Najafgarh,Delhi, India	Sorghum
5	UP-87	*T. asperellum*	KM100821	Manipuri, Uttar Pradesh, India	Pearl millet
5	UP-88	*T. virens*	KM100822	Agra, Uttar Pradesh, India	Pearl millet
5	UP-89	*T. virens*	KM100823	Mathura, Uttar Pradesh, India	Pearl millet
5	UP-90	*T. virens*	KM100837	Mathura, Uttar Pradesh, India	Pearl millet
5	UP-91	*T. brevicompactum*	KM100836	Manipuri, Uttar Pradesh, India	Pearl millet
